# Trichlorido(*N*,*N*′-di-tert-butyl­benzamidinato-κ^2^ *N*,*N*′)silicon

**DOI:** 10.1107/S1600536808010398

**Published:** 2008-04-18

**Authors:** Lu-Dan Lv, Jun-Jun Li, Wei Yang, Chun-Xia Ren, Yu-Qiang Ding

**Affiliations:** a School of Chemical and Materials Engineering, Jiangnan University, 1800 Lihu Road, Wuxi, Jiangsu Province 214122, People’s Republic of China; b College of Pharmacy, GuangDong Pharmaceutical University, Guangzhou, Guangdong Province 510006, People’s Republic of China

## Abstract

In the title mol­ecule, C_15_H_23_Cl_3_N_2_Si, the Si atom is penta­coordinated by two N atoms [Si—N = 1.780 (3) and 1.931 (3) Å] from the benzamidinate ligand and three chloride anions [Si—Cl = 2.0711 (14)–2.1449 (14) Å] in a distorted trigonal-bipyramidal geometry.

## Related literature

For the geometric parameters of related silicon complexes, see: So *et al.* (2006 ▶); Hargittai *et al.* (1983 ▶); Koe *et al.* (1998 ▶); Karsch *et al.* (1998 ▶); Jones *et al.* (2002 ▶).
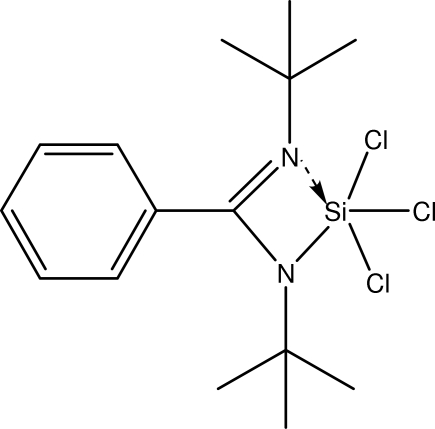


## Experimental

### 

#### Crystal data

C_15_H_23_Cl_3_N_2_Si
*M* *_r_* = 365.80
Triclinic, 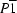

*a* = 6.372 (3) Å
*b* = 10.278 (4) Å
*c* = 14.229 (6) Å
α = 83.222 (6)°
β = 83.227 (6)°
γ = 84.189 (6)°
*V* = 915.3 (7) Å^3^
*Z* = 2
Mo *K*α radiation
μ = 0.56 mm^−1^
*T* = 273 (2) K
0.35 × 0.26 × 0.15 mm

#### Data collection

Bruker SMART CCD area-detector diffractometer
Absorption correction: none
4535 measured reflections
3166 independent reflections
2189 reflections with *I* > 2σ(*I*)
*R*_int_ = 0.028

#### Refinement

*R*[*F* ^2^ > 2σ(*F* ^2^)] = 0.053
*wR*(*F* ^2^) = 0.159
*S* = 0.99
3166 reflections
196 parameters
H-atom parameters constrained
Δρ_max_ = 0.44 e Å^−3^
Δρ_min_ = −0.43 e Å^−3^

### 

Data collection: *SMART* (Bruker, 1998 ▶); cell refinement: *SAINT-Plus* (Bruker, 1998 ▶); data reduction: *SAINT-Plus*; program(s) used to solve structure: *SHELXS97* (Sheldrick, 2008 ▶); program(s) used to refine structure: *SHELXL97* (Sheldrick, 2008 ▶); molecular graphics: *PLATON* (Spek, 2003 ▶); software used to prepare material for publication: *SHELXL97*.

## Supplementary Material

Crystal structure: contains datablocks global, I. DOI: 10.1107/S1600536808010398/cv2396sup1.cif

Structure factors: contains datablocks I. DOI: 10.1107/S1600536808010398/cv2396Isup2.hkl

Additional supplementary materials:  crystallographic information; 3D view; checkCIF report

## Figures and Tables

**Fig. 1.**
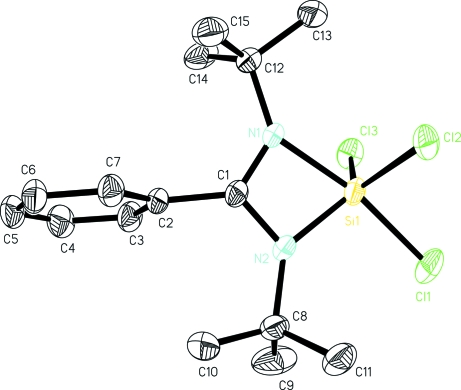
The molecular structure of (I) showing 30% probability displacement ellipsoids and the atom-numbering scheme. H atoms have been omitted for clarity.

## supplementary crystallographic information

### Comment

The discrete electronically neutral mononuclear heteroleptic title silicon(IV) complex, (I), crystallizes in the triclinic space group P-1. The mean plane of  Si1/N1/C1/N2  and phenyl ring C2-C7 form a dihedral angle of 79.1 (1) °. The Si-Cl bond lengths lie in the range 2.0711 (14)-2.1449 (14) Å and agree well with those observed in the related silicon complexes (So *et al.*, 2006; Hargittai *et al.*, 1983; Koe *et al.*, 1998). The N1-C1 bond [1.308 (4) Å] is a typical double bond, while C1-N2 bond [1.368 (4) Å] is intermediate between the double and single C-N bonds. The N1-Si1-N2 angle [70.1 (1) °] in (I) is comparable to that in [PhC(NtBu)_2_]SiCl [68.4 (1) °] (So *et al.*, 2006) and in [MeC(Nipr)_2_]_2_SiCl_2_ [68.8 (1) and 69.0 (1) °] (Karsch *et al.*, 1998). The Si-N bond lengths of 1.780 (3) and 1.931 (3) Å are slightly longer than the Si—N_amide_ bond length in the silicon(IV) complex (C_5_H_3_N-6-Me-2-NSiMe_3_)SiCl_3_ [1.753 (5) Å] (Jones *et al.*, 2002).

### Experimental

All manipulations were carried out in an inert atmosphere of N_2_ using standard Schlenk techniques and in a N_2_ filled glove box. Solvents were dried over and distilled from Na/K alloy prior to use.

PhLi (3.6 ml, 6.48 mmol, 1.8 mol/*L* in cyclohexane/Et_2_O (7:3)) was added to a solution of tBuN=C=NtBu(1.25 ml, 6.48 mmol) in Et_2_O (35 ml) at -78 °C. The solution was raised to ambient temperature and stirred for 1 h. SiCl_4_ (0.8 ml, 6.97 mmol) was added to this solution at -78 °C. The resulting yellow suspension was stirred overnight at ambient temperature. The precipitate was filtered, and the filtrate was concentrated under reduced pressure until colourless crystals of the title compound (1.11 g, 46%) were obtained. *M*.p. 178 °C. Elemental analysis (%) calcd for C_15_H_23_Cl_3_N_2_Si: C 49.24, H 6.34, N 7.66; found: C 49.17, H 6.42, N 7.71. ^1^H NMR (400 MHz, CDCl_3_, 25 °C): δ = 1.18 (s, 18H, tBu), 7.42–7.68 p.p.m. (m, 5H, Ph).

### Refinement

The H atoms were positioned geometrically (C—H 0.93–0.97 Å), and allowed to ride on their parent atoms, with *U*_iso_(H) = 1.2–1.5 *U*_eq_(C).

### Crystal data

**Table:**

C_15_H_23_Cl_3_N_2_Si	*Z* = 2
*M**_r_* = 365.80	*F*(000) = 384
Triclinic, *P*1	*D*_x_ = 1.327 Mg m^−^^3^
*a* = 6.372 (3) Å	Mo *K*α radiation, λ = 0.71073 Å
*b* = 10.278 (4) Å	Cell parameters from 1365 reflections
*c* = 14.229 (6) Å	θ = 2.0–25.0°
α = 83.222 (6)°	µ = 0.56 mm^−^^1^
β = 83.227 (6)°	*T* = 273 K
γ = 84.189 (6)°	Block, colourless
*V* = 915.3 (7) Å^3^	0.35 × 0.26 × 0.15 mm

### Data collection

**Table:**

Bruker SMART CCD area-detector diffractometer	2189 reflections with *I* > 2σ(*I*)
Radiation source: fine-focus sealed tube	*R*_int_ = 0.028
graphite	θ_max_ = 25.0°, θ_min_ = 2.0°
φ and ω scans	*h* = −7→7
4535 measured reflections	*k* = −7→12
3166 independent reflections	*l* = −16→16

### Refinement

**Table:**

Refinement on *F*^2^	Primary atom site location: structure-invariant direct methods
Least-squares matrix: full	Secondary atom site location: difference Fourier map
*R*[*F*^2^ > 2σ(*F*^2^)] = 0.053	Hydrogen site location: inferred from neighbouring sites
*wR*(*F*^2^) = 0.159	H-atom parameters constrained
*S* = 0.99	*w* = 1/[σ^2^(*F*_o_^2^) + (0.102*P*)^2^] where *P* = (*F*_o_^2^ + 2*F*_c_^2^)/3
3166 reflections	(Δ/σ)_max_ < 0.001
196 parameters	Δρ_max_ = 0.44 e Å^−^^3^
0 restraints	Δρ_min_ = −0.43 e Å^−^^3^

### Special details

**Table:**

Geometry. All e.s.d.'s (except the e.s.d. in the dihedral angle between two l.s. planes) are estimated using the full covariance matrix. The cell e.s.d.'s are taken into account individually in the estimation of e.s.d.'s in distances, angles and torsion angles; correlations between e.s.d.'s in cell parameters are only used when they are defined by crystal symmetry. An approximate (isotropic) treatment of cell e.s.d.'s is used for estimating e.s.d.'s involving l.s. planes.
Refinement. Refinement of *F*^2^ against ALL reflections. The weighted *R*-factor *wR* and goodness of fit *S* are based on *F*^2^, conventional *R*-factors *R* are based on *F*, with *F* set to zero for negative *F*^2^. The threshold expression of *F*^2^ > σ(*F*^2^) is used only for calculating *R*-factors(gt) *etc*. and is not relevant to the choice of reflections for refinement. *R*-factors based on *F*^2^ are statistically about twice as large as those based on *F*, and *R*- factors based on ALL data will be even larger.

### Fractional atomic coordinates and isotropic or equivalent isotropic displacement parameters (Å^2^)

**Table:**

	*x*	*y*	*z*	*U*_iso_*/*U*_eq_	
Si1	0.38635 (14)	0.09933 (9)	0.76514 (7)	0.0428 (3)	
Cl1	0.54115 (17)	−0.02445 (10)	0.66177 (8)	0.0732 (4)	
Cl2	0.22531 (16)	−0.05055 (9)	0.84243 (7)	0.0598 (3)	
Cl3	0.67974 (13)	0.13745 (9)	0.80465 (7)	0.0564 (3)	
N1	0.2423 (4)	0.2341 (2)	0.83913 (18)	0.0396 (6)	
N2	0.2521 (4)	0.2228 (3)	0.68974 (18)	0.0443 (7)	
C1	0.1884 (5)	0.3019 (3)	0.7605 (2)	0.0390 (7)	
C2	0.1051 (5)	0.4426 (3)	0.7473 (2)	0.0406 (8)	
C3	0.2489 (6)	0.5342 (3)	0.7133 (3)	0.0536 (9)	
H3	0.3908	0.5063	0.6976	0.064*	
C4	0.1823 (7)	0.6665 (4)	0.7027 (3)	0.0649 (11)	
H4	0.2794	0.7278	0.6807	0.078*	
C5	−0.0281 (8)	0.7075 (4)	0.7249 (3)	0.0675 (12)	
H5	−0.0728	0.7967	0.7177	0.081*	
C6	−0.1716 (6)	0.6183 (4)	0.7572 (3)	0.0596 (10)	
H6	−0.3137	0.6470	0.7716	0.072*	
C7	−0.1065 (5)	0.4847 (3)	0.7686 (2)	0.0499 (9)	
H7	−0.2046	0.4240	0.7906	0.060*	
C8	0.1975 (6)	0.2399 (4)	0.5881 (2)	0.0564 (10)	
C9	0.3937 (9)	0.2735 (6)	0.5218 (3)	0.0974 (18)	
H9A	0.4331	0.3576	0.5330	0.146*	
H9B	0.3638	0.2767	0.4570	0.146*	
H9C	0.5081	0.2074	0.5335	0.146*	
C10	0.0117 (9)	0.3443 (4)	0.5736 (3)	0.0904 (16)	
H10A	−0.1015	0.3288	0.6233	0.136*	
H10B	−0.0376	0.3396	0.5130	0.136*	
H10C	0.0577	0.4300	0.5755	0.136*	
C11	0.1190 (7)	0.1105 (4)	0.5664 (3)	0.0682 (12)	
H11A	0.2315	0.0412	0.5708	0.102*	
H11B	0.0761	0.1215	0.5032	0.102*	
H11C	0.0005	0.0882	0.6115	0.102*	
C12	0.2239 (5)	0.2735 (3)	0.9381 (2)	0.0452 (8)	
C13	0.3108 (7)	0.1583 (4)	1.0033 (3)	0.0732 (12)	
H13A	0.2254	0.0860	1.0053	0.110*	
H13B	0.3077	0.1839	1.0662	0.110*	
H13C	0.4543	0.1319	0.9796	0.110*	
C14	0.3482 (7)	0.3920 (4)	0.9418 (3)	0.0686 (12)	
H14A	0.4918	0.3739	0.9145	0.103*	
H14B	0.3474	0.4087	1.0068	0.103*	
H14C	0.2833	0.4677	0.9064	0.103*	
C15	−0.0083 (6)	0.3052 (4)	0.9733 (3)	0.0659 (11)	
H15A	−0.0616	0.3854	0.9388	0.099*	
H15B	−0.0213	0.3155	1.0400	0.099*	
H15C	−0.0884	0.2348	0.9633	0.099*	

### Atomic displacement parameters (Å^2^)

**Table:**

	*U*^11^	*U*^22^	*U*^33^	*U*^12^	*U*^13^	*U*^23^
Si1	0.0501 (6)	0.0290 (5)	0.0498 (6)	−0.0020 (4)	−0.0022 (4)	−0.0107 (4)
Cl1	0.0790 (7)	0.0593 (7)	0.0827 (8)	0.0118 (5)	0.0007 (6)	−0.0375 (6)
Cl2	0.0756 (7)	0.0345 (5)	0.0695 (6)	−0.0162 (4)	−0.0042 (5)	−0.0006 (4)
Cl3	0.0471 (5)	0.0533 (6)	0.0713 (6)	−0.0056 (4)	−0.0073 (4)	−0.0152 (5)
N1	0.0508 (15)	0.0308 (15)	0.0374 (15)	0.0031 (12)	−0.0052 (12)	−0.0105 (12)
N2	0.0605 (17)	0.0336 (15)	0.0396 (15)	−0.0012 (13)	−0.0024 (13)	−0.0129 (13)
C1	0.0439 (17)	0.0298 (17)	0.0451 (19)	−0.0076 (14)	−0.0032 (14)	−0.0094 (15)
C2	0.053 (2)	0.0281 (17)	0.0425 (18)	−0.0024 (15)	−0.0092 (15)	−0.0083 (14)
C3	0.064 (2)	0.035 (2)	0.063 (2)	−0.0093 (17)	−0.0059 (18)	−0.0084 (17)
C4	0.087 (3)	0.035 (2)	0.075 (3)	−0.017 (2)	−0.016 (2)	−0.002 (2)
C5	0.101 (3)	0.031 (2)	0.073 (3)	0.005 (2)	−0.029 (2)	−0.0103 (19)
C6	0.065 (2)	0.046 (2)	0.068 (3)	0.0146 (19)	−0.015 (2)	−0.0132 (19)
C7	0.056 (2)	0.0355 (19)	0.060 (2)	−0.0060 (16)	−0.0092 (17)	−0.0067 (17)
C8	0.088 (3)	0.045 (2)	0.040 (2)	−0.015 (2)	−0.0090 (18)	−0.0070 (17)
C9	0.136 (5)	0.114 (4)	0.049 (3)	−0.065 (4)	0.010 (3)	−0.008 (3)
C10	0.151 (5)	0.064 (3)	0.063 (3)	0.016 (3)	−0.053 (3)	−0.014 (2)
C11	0.087 (3)	0.061 (3)	0.064 (3)	−0.017 (2)	−0.013 (2)	−0.023 (2)
C12	0.055 (2)	0.042 (2)	0.0395 (18)	0.0009 (16)	−0.0067 (15)	−0.0108 (16)
C13	0.105 (3)	0.066 (3)	0.046 (2)	0.022 (2)	−0.020 (2)	−0.009 (2)
C14	0.091 (3)	0.068 (3)	0.055 (2)	−0.029 (2)	−0.004 (2)	−0.025 (2)
C15	0.068 (3)	0.078 (3)	0.049 (2)	−0.001 (2)	0.0006 (19)	−0.009 (2)

### Geometric parameters (Å, °)

**Table:**

Si1—N2	1.780 (3)	C8—C11	1.544 (5)
Si1—N1	1.931 (3)	C9—H9A	0.9600
Si1—Cl2	2.0711 (14)	C9—H9B	0.9600
Si1—Cl3	2.1005 (14)	C9—H9C	0.9600
Si1—Cl1	2.1449 (14)	C10—H10A	0.9600
N1—C1	1.308 (4)	C10—H10B	0.9600
N1—C12	1.499 (4)	C10—H10C	0.9600
N2—C1	1.368 (4)	C11—H11A	0.9600
N2—C8	1.513 (4)	C11—H11B	0.9600
C1—C2	1.488 (4)	C11—H11C	0.9600
C2—C7	1.383 (5)	C12—C13	1.516 (5)
C2—C3	1.386 (5)	C12—C15	1.520 (5)
C3—C4	1.379 (5)	C12—C14	1.527 (5)
C3—H3	0.9300	C13—H13A	0.9600
C4—C5	1.375 (6)	C13—H13B	0.9600
C4—H4	0.9300	C13—H13C	0.9600
C5—C6	1.363 (6)	C14—H14A	0.9600
C5—H5	0.9300	C14—H14B	0.9600
C6—C7	1.390 (5)	C14—H14C	0.9600
C6—H6	0.9300	C15—H15A	0.9600
C7—H7	0.9300	C15—H15B	0.9600
C8—C9	1.518 (6)	C15—H15C	0.9600
C8—C10	1.531 (6)		
			
N2—Si1—N1	70.14 (12)	C9—C8—C11	110.8 (3)
N2—Si1—Cl2	120.61 (11)	C10—C8—C11	105.1 (3)
N1—Si1—Cl2	94.21 (10)	C8—C9—H9A	109.5
N2—Si1—Cl3	118.03 (10)	C8—C9—H9B	109.5
N1—Si1—Cl3	90.61 (9)	H9A—C9—H9B	109.5
Cl2—Si1—Cl3	119.05 (6)	C8—C9—H9C	109.5
N2—Si1—Cl1	100.24 (10)	H9A—C9—H9C	109.5
N1—Si1—Cl1	169.82 (10)	H9B—C9—H9C	109.5
Cl2—Si1—Cl1	93.66 (6)	C8—C10—H10A	109.5
Cl3—Si1—Cl1	91.20 (6)	C8—C10—H10B	109.5
C1—N1—C12	129.8 (3)	H10A—C10—H10B	109.5
C1—N1—Si1	89.35 (19)	C8—C10—H10C	109.5
C12—N1—Si1	139.8 (2)	H10A—C10—H10C	109.5
C1—N2—C8	128.9 (3)	H10B—C10—H10C	109.5
C1—N2—Si1	94.06 (19)	C8—C11—H11A	109.5
C8—N2—Si1	136.8 (2)	C8—C11—H11B	109.5
N1—C1—N2	105.9 (3)	H11A—C11—H11B	109.5
N1—C1—C2	127.5 (3)	C8—C11—H11C	109.5
N2—C1—C2	126.0 (3)	H11A—C11—H11C	109.5
N1—C1—Si1	56.34 (16)	H11B—C11—H11C	109.5
N2—C1—Si1	49.93 (16)	N1—C12—C13	108.6 (3)
C2—C1—Si1	167.6 (2)	N1—C12—C15	109.8 (3)
C7—C2—C3	119.5 (3)	C13—C12—C15	107.9 (3)
C7—C2—C1	122.9 (3)	N1—C12—C14	111.2 (3)
C3—C2—C1	117.6 (3)	C13—C12—C14	109.3 (3)
C4—C3—C2	120.2 (4)	C15—C12—C14	110.0 (3)
C4—C3—H3	119.9	C12—C13—H13A	109.5
C2—C3—H3	119.9	C12—C13—H13B	109.5
C5—C4—C3	119.8 (4)	H13A—C13—H13B	109.5
C5—C4—H4	120.1	C12—C13—H13C	109.5
C3—C4—H4	120.1	H13A—C13—H13C	109.5
C6—C5—C4	120.5 (4)	H13B—C13—H13C	109.5
C6—C5—H5	119.7	C12—C14—H14A	109.5
C4—C5—H5	119.7	C12—C14—H14B	109.5
C5—C6—C7	120.3 (4)	H14A—C14—H14B	109.5
C5—C6—H6	119.9	C12—C14—H14C	109.5
C7—C6—H6	119.9	H14A—C14—H14C	109.5
C2—C7—C6	119.6 (3)	H14B—C14—H14C	109.5
C2—C7—H7	120.2	C12—C15—H15A	109.5
C6—C7—H7	120.2	C12—C15—H15B	109.5
N2—C8—C9	109.0 (3)	H15A—C15—H15B	109.5
N2—C8—C10	111.9 (3)	C12—C15—H15C	109.5
C9—C8—C10	111.4 (4)	H15A—C15—H15C	109.5
N2—C8—C11	108.6 (3)	H15B—C15—H15C	109.5
